# Effect of a Tea Polyphenol on Different Levels of Exposure of Nicotine and Tobacco Extract on *Streptococcus mutans* Biofilm Formation

**DOI:** 10.3389/froh.2021.737378

**Published:** 2021-12-01

**Authors:** Emily S. Taylor, Grace F. Gomez, Elizabeth A. S. Moser, Brian J. Sanders, Richard L. Gregory

**Affiliations:** ^1^Riley Hospital for Children, Indiana University, Indianapolis, IN, United States; ^2^Biomedical Science, Comprehensive Care, Pathology and Laboratory Medicine, Schools of Dentistry and Medicine, Indiana University, Indianapolis, IN, United States; ^3^Department of Biostatistics, Indiana University, Indianapolis, IN, United States

**Keywords:** biofilm, polyphenol, nicotine, *Streptococcus mutans*, cigarette

## Abstract

**Objective:** The purpose of this study was to compare the effects of different levels of nicotine and tobacco extract exposure on *Streptococcus mutans* biofilm formation and the inhibitory effect of the polyphenol epigallocatechin-3 gallate (EGCG) found in green tea. This study addressed the results of biofilm assays with EGCG and varying relative concentrations of nicotine and tobacco extract consistent with primary, secondary and tertiary levels of smoking exposure. Primary smoking exposure to nicotine has been demonstrated to significantly increase biofilm formation, while EGCG has been demonstrated to reduce *S. mutans* biofilm formation.

**Methods:** *S. mutans* was treated with varying levels of nicotine or cigarette smoke condensate (CSC) concentrations (0–32 mg/ml and 0–2 mg/ml, respectively) in Tryptic Soy broth supplemented with 1% sucrose for different lengths of time simulating primary, secondary and tertiary smoking exposure with and without 0.25 mg/ml EGCG. The amount of total growth and biofilm formed was determined using a spectrophotometric crystal violet dye staining assay.

**Results:** For both nicotine and CSC, primary exposure displayed overall significantly less growth compared to secondary exposure. For nicotine, secondary exposure demonstrated significantly greater growth than tertiary exposure levels. Overall, significantly greater total bacterial growth and biofilm formation in the presence of nicotine and CSC was observed in the absence of EGCG than in the presence of EGCG. However, biofilm growth was not significantly different among different concentrations of CSC.

**Conclusion:** The results of this study help illustrate that nicotine-induced *S. mutans* biofilm formation is reduced by the presence of EGCG. This provides further evidence of the potential beneficial properties of polyphenols.

## Introduction

According to a 2003 World Health Report, dental caries is a disease that poses a significant public health problem and is estimated to affect 60–90% of schoolchildren and most adults in industrialized countries [1]. In the United States alone, an estimated 28% of children are affected by dental caries [2]. Dental caries is perhaps most notably associated with the presence of the bacterial species *Streptococcus mutans* [3]. *S. mutans* grows in two main phases: adherent biofilm and planktonically. The bacterium exhibits cariogenic properties due to its ability to form adherent biofilm, metabolize dietary carbohydrates into lactic acid which causes a drop in oral pH and is tolerant of growth in acidic conditions [3]. Oral biofilms containing *S. mutans* are formed by aggregations of many species of oral bacteria, which attach to hard and soft oral tissues as well as to one another to form bacterial communities. This biofilm is constantly bathed in the human oral cavity by saliva [3]. Another state in which oral microorganisms may be found is that of free-floating, unattached planktonic cells in a fluid environment such as saliva [3].

A current health issue in modern society is that of tobacco use. CDC data published in 2012 states that approximately 42.1 million people (adults aged 18 or older) are current cigarette smokers [4]. Those who smoke receive primary (or first hand) exposure to nicotine and tobacco products. Smoking habits are also affecting younger populations by way of second hand smoke (i.e., secondary nicotine/tobacco exposure). Second hand smoke is defined by the CDC as “sidestream smoke (the smoke released from the burning end of a cigarette) and exhaled mainstream smoke (the smoke exhaled by the smoker) [5].” CDC reports suggest that “an estimated 22 million children aged 3–11 years and 18 million youth aged 12–19 years were exposed to second hand smoke in the United States in 2000 [5].” In addition to second hand smoke, many children are also exposed to third hand smoke (i.e., tertiary nicotine/tobacco exposure), which results when residual tobacco products are deposited onto surfaces. Third hand smoke clings to surfaces such as skin, clothes, furniture, bedding, walls, and vehicles, even long after the smoking in the area has ceased. Exposure to third hand smoke can pose potential health dangers for those who come into contact with it (i.e., inhalation, touching, ingesting), especially children [6].

The metabolic activity and biofilm formation of *S. mutans* is increased in the presence of nicotine (similar to primary smoking exposure) in a dose-dependent fashion, up to 16 mg/ml of nicotine [7]. There has also been a positive link established between increased number of decayed, missing or filled teeth (DMFT) and smoking habits among individuals [8]. In addition, there have been associations shown with children exposed to passive secondhand smoke and increased DMFT scores [9].

A popular beverage of choice for many individuals the world over, is tea. The main antioxidant components in tea belong to a chemical group known as polyphenols. Of the polyphenols in tea, epigallocatechin-3 gallate (EGCG) is the most prevalent [10]. Some research studies illustrate a relationship between people who drink tea and a decrease in caries incidence [11–13]. Proposed mechanisms by which EGCG limits *S. mutans* activity, thereby decreasing caries incidence, is by inhibiting glucosyltransferases (GTF's) which aid in *S. mutans* biofilm formation [11, 12, 14–17]. *S. mutans* may also be negatively affected by EGCG through other mechanisms including inhibition of enolase and lactate dehydrogenase enzymes [16].

This study illustrates the results of total growth and biofilm assays using a standard concentration of EGCG and various nicotine and CSC levels consistent with primary, secondary and tertiary levels of nicotine or tobacco exposure. Primary exposure was obtained by constant 24 h incubation of the bacterium in the presence of the nicotine or tobacco. Secondary exposure was defined for experimental purposes as the exposure of the bacterium to nicotine or tobacco for 1 h. Tertiary exposure was defined as the coating of nicotine or tobacco onto the microtiter plates for 24 h before the bacteria were added to the plates. The purpose of this study was to compare the effects of nicotine and CSC exposure on primary, secondary and tertiary *S. mutans* biofilm formation and the inhibitory effect of EGCG.

## Materials and Methods

### Bacterial Strains, Growth Conditions and Reagents

An overnight culture of *S. mutans* UA159 (ATCC 700610) was grown in Tryptic Soy Broth (TSB, Acumedia, Baltimore, MD) at 37°C in 5% CO_2_. A stock solution of EGCG was dissolved in ETOH (100 mg/ml) and stored at −20°C until time of use. EGCG was diluted with TSB supplemented with 1% sucrose (TSBS) for a final concentration of 0.25 mg/ml [18]. The concentration of 0.25 mg/ml EGCG was determined based on a previous study by Foltz *et al*. which indicated that when EGCG was used alone at this concentration, it demonstrated significant inhibitory growth effects on *S. mutans*, but did not reach toxic exposure levels (J.A. Foltz, personal communication). This concentration of EGCG is also less than what is found in a typical cup of green tea [18].

### Primary Nicotine or CSC Exposure

Serial dilutions of nicotine in TSBS were prepared to yield 0, 0.25, 0.5, 1.0, 2.0, 4.0, 8.0, 16.0 and 32.0 mg/ml nicotine (Sigma-Aldrich Chemical Co., St. Louis, MO) without EGCG, and the same concentrations of nicotine with 0.25 mg/ml of EGCG. 190 μl of TSBS at each nicotine concentration with and without EGCG was aliquoted in quadruplicate into wells of a sterile 96-well flat bottom microtiter plate. 10 μl of a fresh overnight TSB culture of *S. mutans* was added to each well. The microtiter plate was incubated in 5% CO_2_ at 37°C for 24 h and total absorbance (measure of biofilm and planktonic cell growth) was measured in a spectrophotometer (SpectraMax 190; Molecular Devices Inc., Sunnyvale, CA) at 595 nm. Planktonic cells were removed from the biofilm microtiter plate wells (leaving attached biofilm), and 200 μl of 10% formaldehyde was added to each well for 30 min to fix the cells. After 30 min, the formaldehyde was removed and the biofilm cells were washed 3 times with deionized water. 200 μl of 0.5% crystal violet dye was added to each well and the biofilm cells stained for 30 min. The wells were washed 3 times and 200 μl of 2-isopropanol was placed in each well for 1 h to lyse the cells and extract the crystal violet [7]. The plates were read in a spectrophotometer at 490 nm to measure biofilm formation including extracellular polysaccharide production and biofilm cell mass.

The same protocol as above was followed to examine the effects of CSC (40 mg/ml particulate material, 940 μg nicotine/ml; Murty Pharmaceuticals, Lexington, KY) diluted to 0, 0.25, 0.5, 1.0, and 2.0 mg/ml of CSC in TSBS. Each experiment was repeated a total of 3 times. Controls included biofilms of *S. mutans* without nicotine/CSC and with 0.25 mg/ml of EGCG or without EGCG.

### Secondary Nicotine/CSC Exposure

To simulate secondary nicotine exposure, *S. mutans* biofilm with and without 0.25 mg/ml EGCG and TSBS was established by incubating *S. mutans* for 16 h in 5% CO_2_ at 37°C. The media was removed and nicotine (0–32 mg/ml) or CSC (0–2 mg/ml) in TSB was added to corresponding wells at 37°C for 1 h. The nicotine/tobacco dilutions were removed and fresh TSBS with EGCG was added for another 16 h before crystal violet staining was performed as above.

### Tertiary Nicotine/CSC Exposure

A representation of third hand smoke involved incubation of nicotine (0–32 mg/ml) or CSC (0–2 mg/ml) dilutions in TSBS (without *S. mutans* or EGCG) at 37°C for 24 h to coat the microtiter plate with nicotine/CSC. After 24 h, the nicotine/CSC dilutions were removed from the wells, and 190 μl of fresh TSBS with and without 0.25 mg/ml EGCG was added, along with 10 μl of an overnight culture of *S. mutans*. The plates were incubated for 24 h in 5% CO_2_ at 37°C and the biofilm was stained with crystal violet as described earlier.

### Statistical Analysis

For both nicotine and CSC, a three-way ANOVA was used to compare the effects of polyphenol exposure (with and without 0.25 mg/ml of EGCG), concentration (nicotine: 0, 0.25, 0.5, 1.0, 2.0, 4.0, 8.0, 16.0, 32.0 mg/ml or CSC: 0, 0.25, 0.5, 1.0, 2.0 mg/ml), and nicotine exposure type (primary, secondary, or tertiary) on total growth and biofilm. Pair-wise comparisons were made using Tukey's method to control the overall significance level at 5%. Prior to the analyses, the distributions of the measurements were examined for violations of ANOVA assumptions. Due to non-normality of the data, rank transformations were performed prior to the analyses. Significance was defined as *p* ≤ 0.05.

## Results

Overall, significantly greater biofilm growth (*p* < 0.05) in the presence of primary, secondary and tertiary nicotine and CSC exposure levels was observed in the absence of EGCG than in the presence of EGCG. However, biofilm growth was not significantly different among the different concentrations of CSC. Significantly greater total bacterial growth was present in the absence of EGCG than in the presence of EGCG. For both nicotine and CSC, primary exposure displayed overall significantly less growth compared to secondary exposure. For nicotine, secondary exposure demonstrated significantly greater growth than tertiary exposure levels.

### Primary Exposure of Nicotine and CSC

#### Biofilm

**Figure 1:**
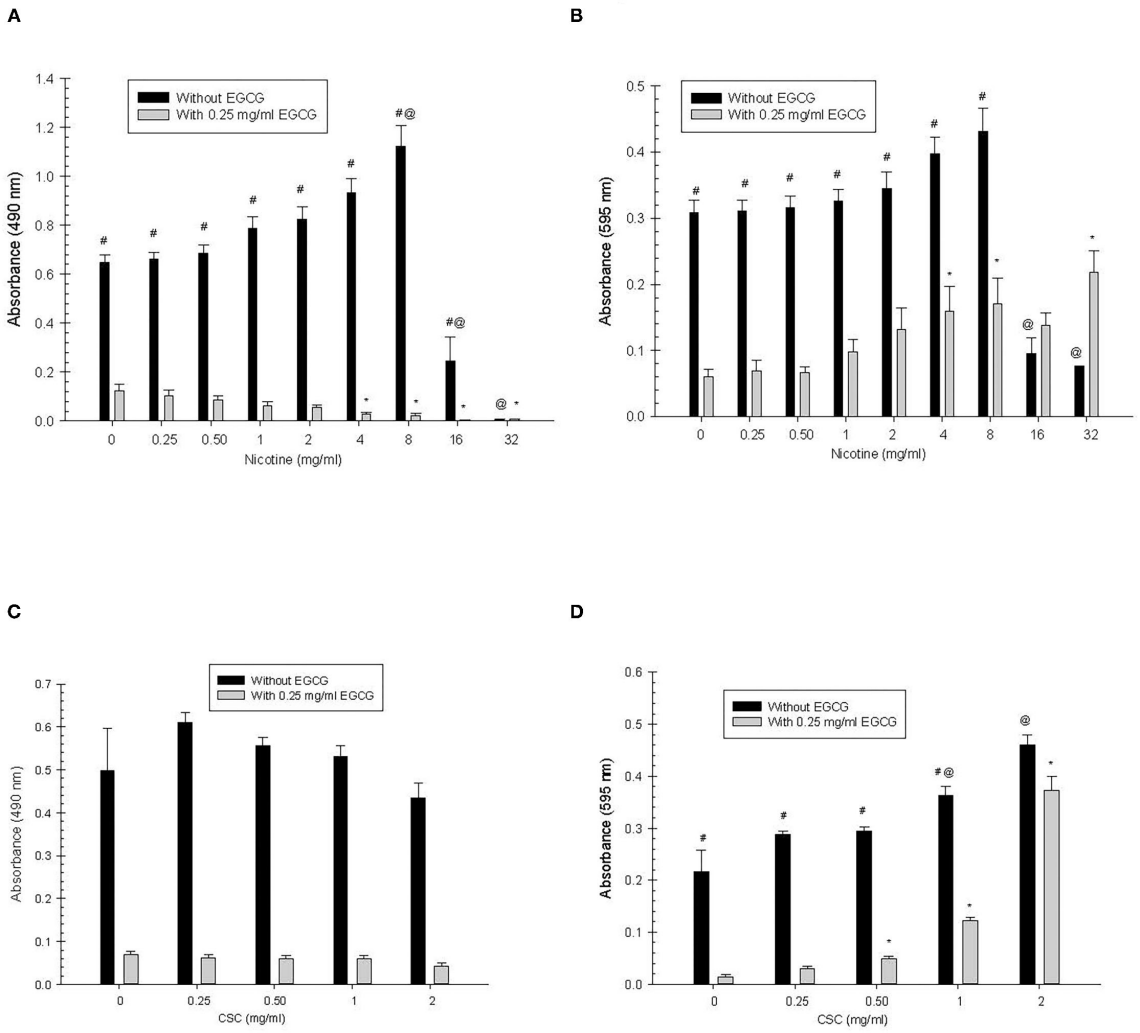
Primary *S. mutans* biofilm **(A,C)** and total absorbance **(B,D)** nicotine/CSC exposure effects. ^#^Indicates significant differences between growth with 0.25 mg/ml of EGCG and without EGCG. * and @ indicates significant differences between 0 mg/ml nicotine/CSC and other concentrations with 0.25 mg/ml of EGCG and without EGCG, respectively.

*Nicotine:* For primary nicotine exposure, there was significantly greater biofilm growth in the absence of EGCG compared to when EGCG was present, for all concentrations of nicotine (*p* < 0.0001) except 32 mg/ml, where there was no significant difference (Figure 1). Growth both in the absence and presence of EGCG was greater at 0 mg/ml compared to 16 and 32 mg/ml nicotine (*p* < 0.0001). In the presence of EGCG, growth at 0 mg/ml was greater than at 4 mg/ml (*p* = 0.0002) and 8 mg/ml nicotine (*p* < 0.0001). In the absence of EGCG, growth at 0 mg/ml was less than at 8 mg/ml nicotine (*p* = 0.0165).

*CSC:* For primary CSC exposure there was significantly greater biofilm growth in the absence of EGCG compared to when EGCG was added (*p* < 0.0001), but there were no significant differences in biofilm growth for the different concentrations of CSC.

#### Total Absorbance

*Nicotine:* For primary nicotine exposure, growth in the absence of EGCG was significantly greater than growth in the presence of EGCG (*p* < 0.0001), except at concentrations of 16 and 32 mg/ml of nicotine, where there was no significant difference. In the presence of EGCG, growth at concentrations of 4, 8, and 32 mg/ml nicotine was significantly greater than at 0 mg/ml nicotine (*p* ≤ 0.0051). In the absence of EGCG, growth at concentrations 16 and 32 mg/ml nicotine was significantly less than at 0 mg/ml nicotine (*p* < 0.0001).

*CSC:* For primary CSC exposure, growth in the absence of EGCG was significantly greater than growth in the presence of EGCG (*p* < 0.0001), except at the concentration of 2 mg/ml CSC, where there was no significant difference. In the presence of EGCG, growth at concentrations of 0.5, 1, and 2 mg/ml CSC was significantly greater than at 0 mg/ml CSC (*p* < 0.0001). In the absence of EGCG, growth at 1 and 2 mg/ml was significantly greater than at 0 mg/ml (*p* < 0.0001).

### Secondary Exposure of Nicotine and CSC

#### Biofilm

**Figure 2:**
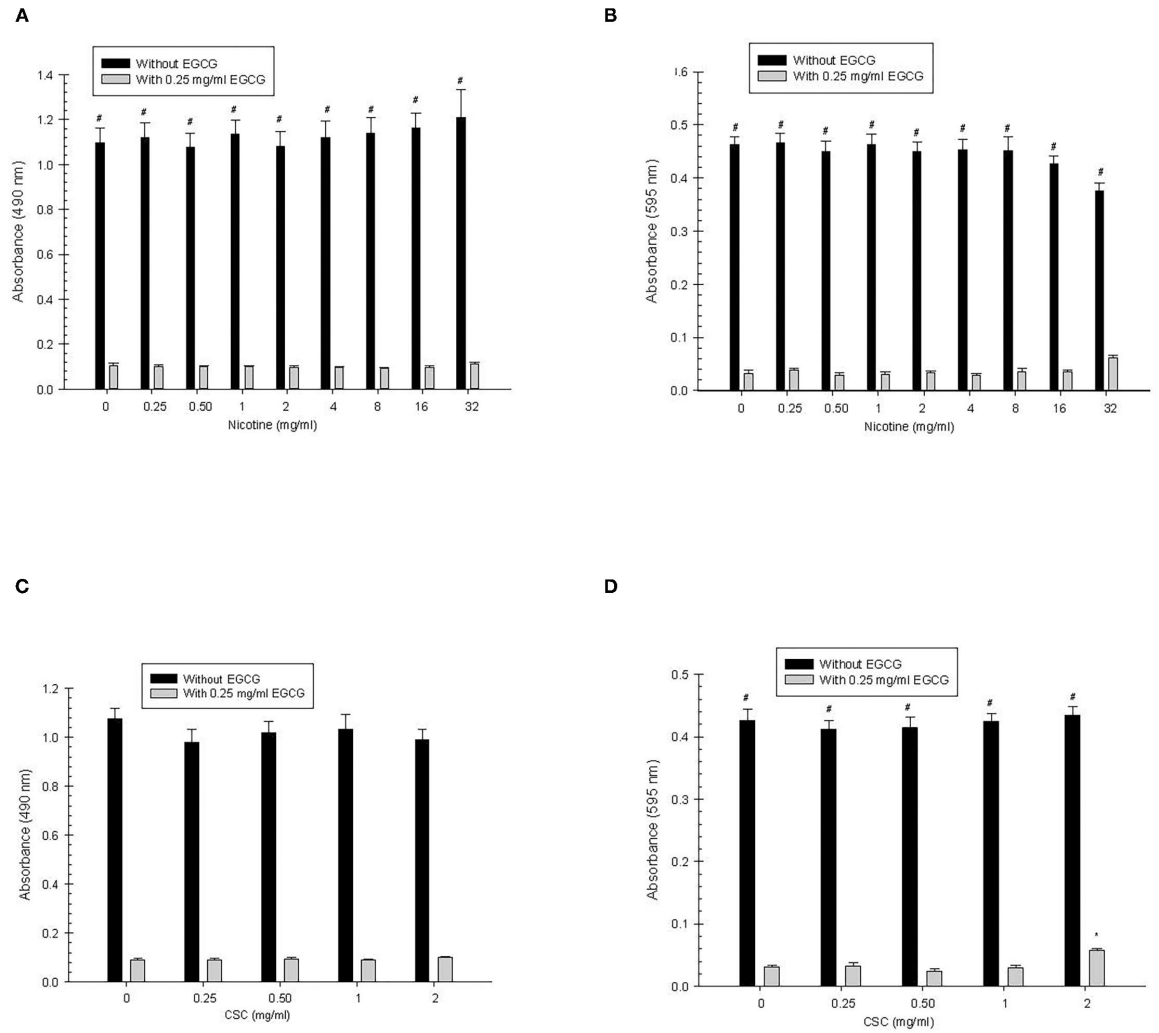
Secondary *S. mutans* biofilm **(A,C)** and total absorbance **(B,D)** nicotine/CSC exposure effects. ^#^Indicates significant differences between growth with 0.25 mg/ml of EGCG and without EGCG. * and @ indicates significant differences between 0 mg/ml nicotine/CSC and other concentrations with 0.25 mg/ml of EGCG and without EGCG, respectively.

*Nicotine:* Overall, there was significantly greater biofilm growth in the absence of EGCG than in the presence of EGCG (*p* < 0.0001) for all concentrations of nicotine (Figure 2). There were no significant differences in biofilm growth for the different concentrations of nicotine.

*CSC:* Overall, biofilm growth in the absence of EGCG was greater (*p* < 0.0001) than with EGCG, but there was no significant difference when comparing biofilm growth with and without EGCG at each of the CSC concentrations. In addition, there were no significant differences in biofilm growth for the different concentrations of CSC.

#### Total Absorbance

*Nicotine:* For each concentration of nicotine, there was significantly greater total growth in the absence of EGCG compared with the presence of EGCG (*p* < 0.0001). There were no significant differences in total absorbance for the different concentrations of nicotine.

*CSC:* For each concentration of CSC, there was significantly greater growth in the absence of EGCG compared with the presence of EGCG (*p* < 0.0001). In the presence of EGCG, growth at 2 mg/ml CSC was greater than 0 mg/ml CSC, however, there were no additional significant differences observed among the various CSC concentrations.

### Tertiary Exposure of Nicotine and CSC

#### Biofilm

**Figure 3:**
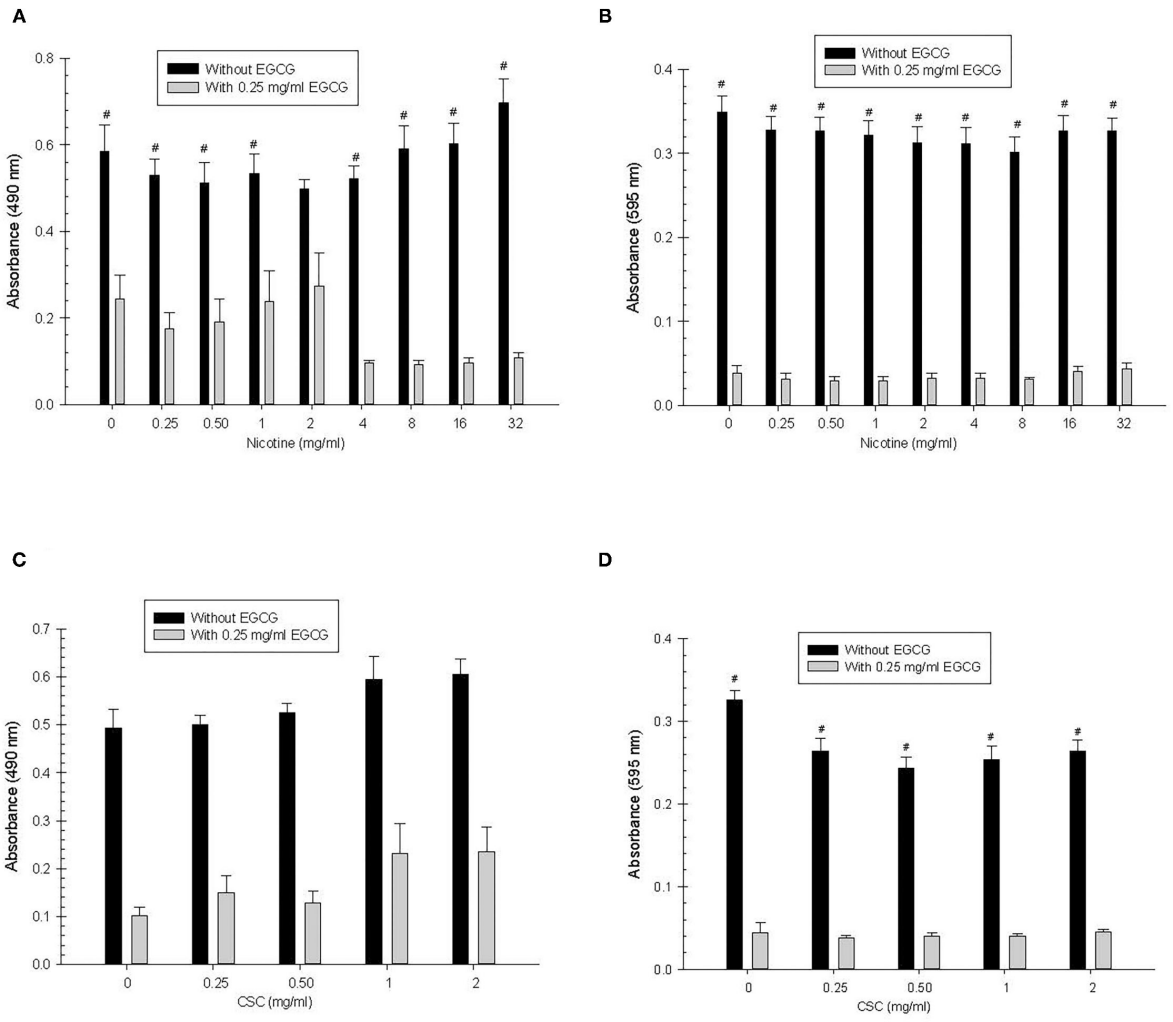
Tertiary *S. mutans* biofilm **(A,C)** and total absorbance **(B,D)** nicotine/CSC exposure effects. ^#^Indicates significant differences between growth with 0.25 mg/ml of EGCG and without EGCG. * and @ indicates significant differences between 0 mg/ml nicotine/CSC and other concentrations with 0.25 mg/ml of EGCG and without EGCG, respectively.

*Nicotine:* There was significantly greater biofilm growth in the absence of EGCG compared with the presence of EGCG for all nicotine concentrations (*p* ≤ 0.004) except at 2 mg/ml nicotine (Figure 3). There were no significant differences in biofilm growth between the different concentrations of nicotine.

*CSC*: Overall, biofilm growth in the absence of EGCG was greater (*p* < 0.0001) than with EGCG, but there was no significant difference when comparing growth with and without EGCG at each of the individual CSC concentrations. Furthermore, there was no significant difference in biofilm growth for the different concentrations of CSC.

#### Total Absorbance

*Nicotine:* For each concentration of nicotine, there was significantly greater total growth in the absence of EGCG compared with the presence of EGCG (*p* < 0.0001) for all concentrations of nicotine. There were no significant differences in total absorbance for the different concentrations of nicotine.

*CSC:* For each concentration of CSC, there was significantly greater total growth in the absence of EGCG compared with the presence of EGCG (*p* < 0.0001). However, there were no significant differences in total absorbance between the different concentrations of CSC.

## Discussion

Data gathered from this study illustrates how growth of *S. mutans* is impacted by primary, secondary and tertiary nicotine/CSC exposure in the presence of the major polyphenol in tea. Overall, biofilm and total absorbance growth were decreased in the presence of EGCG, compared to when ECGC was absent. Earlier preliminary work by Foltz et al. (unpublished data) indicated a significant dose dependent effect of EGCG on *S. mutans* growth and biofilm formation. Specifically, concentrations above 0.5 mg/ml totally ablated *S. mutans* growth and biofilm formation while 0.25 mg/ml partially reduced both growth and biofilm formation. EGCG may cause inactivation of critical surface factors such as enzymes and other proteins and lipids.

For primary biofilm growth with nicotine in the absence of EGCG, at the highest concentrations of nicotine (16 and 32 mg/ml), there was significantly decreased growth compared to the 0 mg/ml control. This is due to the high levels of nicotine inhibiting bacterial growth [7]. Similar effects are seen in the presence of EGCG with nicotine at 4, 8, 16, and 32 mg/ml displaying less growth than at 0 mg/ml. This may be due to these concentrations of nicotine inhibiting bacterial growth, compounded additionally by the inhibitory effects of EGCG. In the absence of ECGC, there is also greater growth observed at 8 mg/ml compared to 0 mg/ml nicotine, supporting evidence for nicotine's ability to promote bacterial growth up to this concentration.

For primary nicotine exposure in the presence of EGCG, total growth at 4, 8, and 32 mg/ml nicotine was greater than at 0 mg/ml. One explanation for this could be that nicotine is potentiating bacterial planktonic growth and subverting the effects of EGCG at these concentrations. EGCG seems to inhibit biofilm growth more efficiently than planktonic growth at primary exposure levels. In the absence of EGCG, total growth at 16 and 32 mg/ml was less than at 0 mg/ml. This is due to the inhibitory effects of nicotine on bacterial growth at high concentrations observed previously [7]. For primary CSC exposure, there was significantly greater growth without EGCG than with EGCG for all concentrations except at 2 mg/ml CSC. This may be due to higher CSC concentrations overcoming the effects of EGCG. In the presence of EGCG, 0.5, 1, and 2 mg/ml of CSC demonstrated greater growth than at 0 mg/ml (*p* < 0.0001). In the absence of EGCG, growth at 1 and 2 mg/ml CSC was greater than at 0 mg/ml. This may be again due to the effects of CSC potentiating bacterial growth and overcoming the inhibitory effects of EGCG.

Overall, secondary and tertiary nicotine and CSC exposure stimulated greater biofilm and total growth in the absence of EGCG compared with the presence of EGCG. This provides evidence for the strong inhibitory effects of EGCG on *S. mutans* biofilm and total growth. Nicotine and CSC both appear to enhance biofilm and total growth of *S. mutans*, providing further evidence for smoking as a contributing factor to caries incidence. A limitation of this study is that the levels of nicotine were not measured in the secondary and tertiary exposures, but it is clear that the relative nicotine concentration was primary > secondary > tertiary exposure. The crystal violet biofilm staining assays used have been used extensively by this laboratory and correlate well with viable bacterial count assays [3, 7]. The growth enhancement seen with CSC and nicotine is inhibited when the polyphenol EGCG is added. Although the antimicrobial mechanism of EGCG is unknown, it is speculated that the bacterial growth inhibition may be due to inactivation of critical surface factors such as enzymes, other proteins and lipids leading to reduced uptake of nutrients or production of glucan.

In addition, there is some synergistic interaction between EGCG and nicotine as biofilm is reduced with a constant concentration of EGCG (0.25 mg/ml) and increasing nicotine concentration. However, total growth of *S. mutans* was increased under similar conditions suggesting that EGCG was able to reduce biofilm formation while increasing planktonic growth. This may be caused by down regulation of biofilm specific receptors causing more cells to detach from the biofilm and enter the planktonic phase.

Limitations of this study include the use of one strain of *S. mutans*, one time point of inhibition and one analytical technique, however, the crystal violet biofilm staining assay used correlates well with bacterial viability plating data. In addition, it is not possible to directly measure actual nicotine concentrations in the secondary or tertiary treatments, however, they are clearly less than the primary treatment and the tertiary is less than the secondary treatment.

Future studies should examine the effects of polyphenols in the presence of CSC/nicotine and caries incidence *in vivo*, at primary, secondary and tertiary exposure levels. Further investigation could also evaluate the bioavailability of polyphenols and if variations in the concentration of polyphenols have a significant impact on *S. mutans* growth. Additional research should examine other types of polyphenols and determine their effects on *S. mutans* growth in the presence of CSC and nicotine. EGCG may be beneficial in the diet of children and adults exposed to nicotine, particularly those exposed to secondary and tertiary exposure.

## Data Availability Statement

The raw data supporting the conclusions of this article will be made available by the authors, without undue reservation.

## Author Contributions

ET provided substantial contributions to acquisition, analysis and interpretation of data, and drafting the article. RG provided substantial contributions to the study design, acquisition, analysis and interpretation of data, critical revision of the article, and final approval of the version to be published. GG and BS provided substantial contributions to interpretation of data and drafting the article. EM provided substantial contributions to the study design, analysis, and interpretation of data.

## Funding

This work was supported, in part, by the Indiana University School of Dentistry Graduate Student Research Fund.

## Conflict of Interest

The authors declare that the research was conducted in the absence of any commercial or financial relationships that could be construed as a potential conflict of interest.

## Publisher's Note

All claims expressed in this article are solely those of the authors and do not necessarily represent those of their affiliated organizations, or those of the publisher, the editors and the reviewers. Any product that may be evaluated in this article, or claim that may be made by its manufacturer, is not guaranteed or endorsed by the publisher.
